# A deep learning approach to mild cognitive impairment detection from electroencephalogram signals

**DOI:** 10.1590/1980-5764-DN-2025-0364

**Published:** 2026-08-24

**Authors:** Hemlata Sandip Ohal, Shamla Mantri

**Affiliations:** 1 Dr. Vishwanath Karad MIT World Peace University, School of Computer Science and Engineering Department of Computer Engineering and Technology, Pune, Maharashtra, India.

**Keywords:** Cognitive Dysfunction, Machine Learning, Electroencephalography, Disfunção Cognitiva, Aprendizado de Máquina, Eletroencefalografia

## Abstract

Mild cognitive impairment (MCI) is an important condition that may progress to Alzheimer disease (AD) or other types of dementia. If MCI can be detected early, timely interventions may be implemented. Traditional diagnostic methods rely on neuropsychological tests and imaging studies but have limitations in terms of efficiency and accessibility. Therefore, electroencephalogram (EEG)-based deep learning approaches represent promising developments that may offer new frameworks for the early diagnosis of MCI.

**Objective:**

This study aimed to develop a long short-term memory (LSTM)-based architecture for classifying MCI using EEG signals. The temporal characteristics of EEG signals may reveal meaningful information that improves classification performance.

**Methods:**

A publicly available EEG dataset comprising 27 subjects (11 MCI; 16 normal) was used. Raw EEG signals were preprocessed using band-pass filtering, Independent Component Analysis, and segmentation. The 64-node LSTM model was trained using processed EEG segments for binary classification. The model was evaluated using standard performance metrics.

**Results:**

The proposed LSTM-based model achieved an accuracy of 98.14% for MCI *versus* normal classification, outperforming conventional algorithms such as K-Nearest Neighbors (KNN) and Support Vector Machine (SVM). The model also demonstrated high precision (99.21%), recall (98.69%), and F1-score (98.95%). The confusion matrix showed 4,774 normal and 3,257 MCI segments correctly classified, with very few misclassifications, highlighting its strong discriminative capability.

**Conclusion:**

This study highlights the potential of LSTM networks for early MCI detection. The findings suggest that deep learning-driven EEG analysis may be a valuable tool for noninvasive and scalable cognitive health assessments.

## RESUMO.

O comprometimento cognitivo leve (*Mild Cognitive Impairment* – MCI) é uma condição importante que pode evoluir para doença de Alzheimer (DA) ou outros tipos de demência. Se o MCI for detectado precocemente, intervenções oportunas poderão ser implementadas. Os métodos diagnósticos tradicionais baseiam-se em testes neuropsicológicos e estudos de imagem, mas apresentam limitações quanto à eficiência e à acessibilidade. Nesse contexto, abordagens de *deep learning* baseadas em eletroencefalograma (EEG) representam avanços promissores, capazes de oferecer novos modelos para o diagnóstico precoce do MCI.

**Objetivo:**

Este estudo teve como objetivo desenvolver uma arquitetura baseada em memória de longo e curto prazo (*Long Short-Term Memory* – LSTM) para a classificação de MCI utilizando sinais de EEG. Artefatos presentes nos dados de EEG podem revelar propriedades temporais que possibilitam aprimorar a classificação.

**Métodos:**

Foi utilizado um conjunto de dados público de EEG composto por 27 indivíduos (11 com MCI; 16 normais). Os sinais brutos de EEG foram pré-processados por meio de filtragem passa-faixa, Análise de Componentes Independentes (*Independent Component Analysis* – ICA) e segmentação. O modelo LSTM com 64 nós foi treinado utilizando segmentos processados de EEG para classificação binária. O desempenho do modelo foi avaliado por métricas padrão.

**Resultados:**

O modelo proposto baseado em LSTM alcançou acurácia de 98,14% para a classificação entre MCI e indivíduos normais, superando algoritmos convencionais, como *K-Nearest Neighbors* (KNN) e *Support Vector Machine* (SVM). O modelo também apresentou elevada precisão (99,21%), recall (98,69%) e escore F1 (98,95%). A matriz de confusão demonstrou 4.774 segmentos normais e 3.257 segmentos com MCI classificados corretamente, com número muito reduzido de classificações incorretas, evidenciando forte capacidade discriminativa.

**Conclusão:**

Este estudo destaca o potencial das redes LSTM para a detecção precoce de MCI. Os achados sugerem que a análise de EEG orientada por *deep learning* pode constituir uma ferramenta valiosa para avaliações da saúde cognitiva não invasivas e escaláveis.

## INTRODUCTION

 Mild cognitive impairment (MCI) indicates a subtle yet noticeable decline in cognitive functions, such as memory and reasoning abilities. It significantly impacts individuals’ daily lives, affecting their ability to perform complex tasks, make decisions, and maintain social relationships. MCI often serves as an intermediate phase between normal cognitive aging and dementia, particularly Alzheimer disease (AD). 

 According to statistics from the World Health Organization (WHO), dementia affects approximately 55 million people globally, with nearly 10 million new diagnoses each year. Although these data focus on dementia, they highlight the importance of early detection and intervention for MCI, which may progress to dementia in the future. Existing methods for diagnosing MCI use multiple sources of information to accurately identify cognitive decline. 

### Related work

 Recent advances in the application of machine learning (ML) and deep learning (DL) for the diagnosis of MCI have shown promising results, although certain limitations persist. One study examined the classification of subjects with MCI and cognitively normal individuals using resting-state functional magnetic resonance imaging (fMRI) data and Support Vector Machine (SVM) and Random Forest (RF) techniques. The reported accuracy ranged from 78 to 90%^1^. 

 Another study applying a Gaussian Naive Bayes classifier achieved an accuracy of 92.14%, further suggesting that digital serious games represent innovative approaches for the detection and screening of cognitive impairment^2^. 

 A method for detecting MCI using gait analysis and RF models achieved an accuracy of 85.50%. The models were evaluated using motion-capture data collected from participants during walking tests^3^. Another study utilized ML models to process data from environmental sensors used to monitor transitions in home activities and walking speed. The SVM and RF models demonstrated strong performance, with an area under the curve (AUC) of 0.97^4^. 

 A study focused on the classification of early and late MCI subtypes using ML models based on data extracted from multimodal MRI demonstrated a mean accuracy of 70% using an ensemble model called AdaBoost^5^. Another novel approach for differentiating participants with MCI from neurologically typical subjects involved the use of functional near-infrared spectroscopy (fNIRS). The proposed representation-based transfer learning method achieved an accuracy improvement of 95.81% by leveraging pretrained convolutional neural network (CNN) models with limited-duration measurements^6^. 

 However, several challenges remain in the application of ML/DL methods for MCI detection. Most available data have been collected from studies with small sample sizes, limiting the generalizability of findings to different populations. Larger datasets could improve the robustness and precision of these models^7^. 

 Long short-term memory (LSTM) networks, a type of recurrent neural network (RNN), were developed to mitigate the exploding and vanishing gradient problems commonly observed in traditional RNNs^8^. A major advancement of LSTM networks is their ability to preserve long-term dependencies in sequential data, enabling their use in tasks involving sequence and/or temporal information^9^. LSTM architectures are currently employed in several state-of-the-art machine learning applications, particularly those in which the temporal dimension of the data is essential. These applications include, but are not limited to, speech and handwriting recognition, sentiment analysis, and NLP tasks such as machine translation^10^. 

 Electroencephalogram (EEG) signal data constitute a multivariate time series representing brain activity over time. These data are non-stationary, highly dynamic, and often noisy, reflecting rapid changes in neural activity. Time-series analysis techniques are essential for extracting meaningful patterns and features from EEG signals. LSTM networks are highly effective for analyzing EEG time-series data because of their ability to capture complex temporal relationships and model nonlinear patterns. 

 This paper is organized as follows: Section II presents the proposed framework for MCI *versus* normal classification, including detailed descriptions of data acquisition, data preprocessing, and the proposed DL approach. This section concludes with the performance parameters used to assess the model. Section III presents the results obtained, followed by Section IV, which discusses these findings and compares them with previous studies. 

### Proposed framework

**Figure 1:**
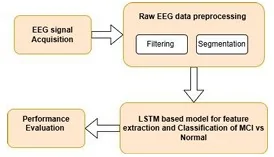
Proposed framework for mild cognitive impairment detection. Source: Created by the authors.

 This study presents a framework for MCI diagnosis based on LSTM networks utilizing EEG data. The design of the proposed framework for MCI detection, as shown in Figure 1, comprises four primary components:

- EEG data acquisition,
- Data preprocessing, which involves filtering the collected EEG data using both low-pass and highpass filters, followed by segmentation,
- Design of the LSTM-based framework for feature extraction and identification of subjects with MCI; and
- Performance assessment.

 A comprehensive explanation of each major component of the framework is provided below: 

 The proposed DL framework introduces several key differences compared with existing methods for MCI detection using EEG signals. Unlike traditional ML models, such as K-Nearest Neighbors (KNN), SVM, and RF, which rely heavily on handcrafted spectral or statistical features, the proposed approach directly processes preprocessed EEG segments. This enables the LSTM network to capture temporal dependencies within the data and learn discriminative patterns that are often overlooked when relying solely on predefined features. 

 Compared with previous EEG-based DL studies that employed larger LSTM architectures with 128 or even, 1,024 nodes, the proposed framework adopts a more compact architecture with 64 nodes. Supported by dropout and dense layers, this structure not only achieves higher accuracy but also minimizes computational complexity and reduces the risk of overfitting. Consequently, the model is more efficient and better suited for small-scale EEG datasets, in which excessively complex networks may struggle to generalize effectively. 

 Furthermore, the preprocessing pipeline combines filtering, independent component analysis (ICA) for artifact removal, and segmentation into six-second windows, resulting in 8,100 temporal sequences. This strategy ensures that the input to the LSTM is cleaner and more representative of the non-stationary and dynamic nature of EEG signals. In contrast, many previous studies relied on longer unsegmented signals or limited feature extraction methods, restricting temporal resolution and often resulting in lower model performance. 

### Data acquisition

 This study utilized a publicly available dataset comprising 27 participants: 11 with MCI and 16 healthy individuals, selected from a previous study^11^. “Participants in this study were recruited from patients undergoing treatment at the Cardiac Catheterization Units of Sina and Nour Medical Centers, Iran. Furthermore, the study received ethical approval from the Research and Technology Division at Isfahan University of Medical SciencesIUMS”^11^. 

 All participants provided written informed consent. To evaluate MCI, a neuropsychiatric interview was conducted according to the Peterson criteria. MCI classification was validated using mini-mental state examination (MMSE) scores. In this dataset, the mean MMSE score for participants with MCI was 27.6 ± 0.9, whereas cognitively normal subjects demonstrated a higher mean score of 29.0 ± 0.8. This distinction is consistent with the expected differences in cognitive performance between the two groups. The Neuropsychiatry Unit Cognitive Assessment Tool (NUCOG) was used as a dependent variable to further substantiate the MCI diagnosis. Table 1 summarizes the demographic characteristics of the dataset. 

**Table 1:** Demographic information of the Isfahan University of Medical Sciences dataset.

Characteristic	MCI (mean value±SD)	Control (mean value±SD)	p-value
Age (years)	66.4±04.6	65.3±03.9	0.4
Education (years)	10.3±03.8	11.1±03.0	0.4
Mini-Mental State Examination scores	27.6±0.9	29.0±0.8	<0.001
Neuropsychiatry Unit Cognitive Assessment score	82.4±3.6	91.1±3	<0.001

Abbreviations: MCI, mild cognitive impairment; SD, standard deviation.

Source: Adapted from Kashefpoor et al.^11^

**Figure 2:**
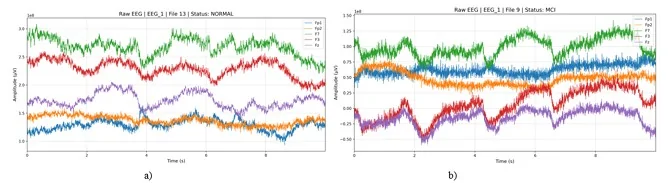
Unprocessed electroencephalogram signal recordings of (A) a normal subject and (B) an individual with mild cognitive impairment. (A) shows the unprocessed electroencephalogram signals plotted for a normal control subject. (B) shows the unprocessed electroencephalogram signals plotted for subjects with mild cognitive impairment. Both plots display only the signals collected from the Fp1, Fp2, F3, F7, and Fz electrodes over a duration of 5 seconds. Source: Created by the authors.

Figure 2 show the raw electroencephalogram signals plotted for a normal control subject and for subjects with mild cognitive impairment, respectively. 

### Data preprocessing

 Raw EEG signal data were obtained from the dataset and preprocessed for artifact removal. Xci and Xpi represent the raw EEG data in European Data Format (EDF) for subject i. In this study, a band-pass filter with a range of 0-60 Hz was applied. A notch filter was also used to specifically eliminate power-line interference at 50 or 60 Hz without disrupting neighboring frequencies. Together, these filters improved the signal-to-noise ratio, reduced artifacts, and standardized the recordings. 

 One of the primary applications of ICA in EEG preprocessing is artifact removal, including ocular artifacts, muscle movements, electrocardiogram (ECG) interference, and line noise. ICA identifies statistically independent components corresponding to these artifacts and isolates them from true brain activity^12,13^. After filtering, ICA was applied to the signals. 

 Segmentation addresses the non-periodic and non-stationary nature of EEG signals while preserving representative information from each data segment. It also helps manage large dataset sizes and reduces computational requirements. Therefore, the 30-minute EEG recordings were segmented into smaller segments of 6 seconds^14^. 

 Segmentation of the cleaned signal into fixed-length windows of duration T seconds is defined as: 


(1) $$S(t,c^{\prime })=\{X_{\mathrm{clean}}(t,c^{\prime }),X_{\mathrm{clean}}(t+T,c^{\prime }),\ldots \}$$

Where t is the time index and c’ is the channel index of preprocessed and cleaned EEG input, representing as X_clean_. The total signal length per subject is given by: 


(2) $$L_{\mathrm{seg}}=f_{s}\cdot T_{\mathrm{seg}}$$

Where f_s_ is the sampling frequency and T_seg_ is the segment duration. 

 After segmentation, a total of 8,100 segments were generated across all subjects, resulting in 8,100 temporal sequences for input into the classification module. 

### Deep learning approach

**Figure 3:**
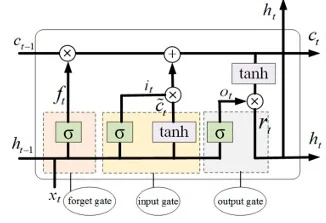
Basic long short-term memory cell framework^16^. Source: Reproduced from Smagulova and James^16^.

 An LSTM network comprises specialized components known as memory cells. Each of these components consists of three essential sections: input, output, and forget gates, as shown in Figure 3 ^15^. Cell operations are regulated through these gates to control the flow of data into, out of, or within the cell. Data entry is controlled by an input gate that determines which part of the input is allowed into the memory cell for storage. The output gate regulates the portion of the memory content that affects other units in the system, whereas the forget gate governs the data to be discarded from the cell’s memory^16^. 

**Figure 4:**
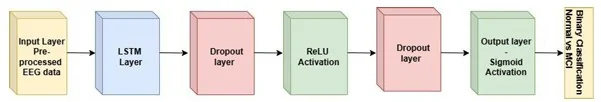
Long short-term memory-based model for mild cognitive impairment vs. normal classification. Source: Created by the authors.

 The proposed LSTM-based model for MCI vs. normal classification is illustrated in Figure 4. Several layers are utilized to capture temporal patterns, introduce nonlinearity, and prevent overfitting in the development of the sequential model. 

#### 1. Input Layer

 The input consists of a sequence of length T with C channels: 


(3) $$X=[x_{1},x_{2},\ldots ,x_{T}],x_{t}\in R^{C}$$

#### 2. LSTM Layer

 The LSTM layer processes the input data sequentially and outputs a final hidden state of size 64 


(4) $$h_{t},c_{t}=\mathrm{LSTM}(x_{t},h_{t-1},c_{t-1})$$

Final output: 


(5) $$h_{\mathrm{final}}=h_{T},h_{\mathrm{final}}\in R^{64}$$

#### 3. Dropout

 Dropout is applied to the LSTM output: 


(6) $$h_{\mathrm{drop}}=\mathrm{Dropout}(h_{\mathrm{final}},p=0.3)$$

#### 4. Dense Layer 1

 The dropped-out hidden state is transformed into a 32-dimensional representation using a dense layer with rectified linear unit (ReLU) activation: 


(7) $$a_{1}=\mathrm{ReLU}(W_{1}h_{\mathrm{drop}}+b_{1}),a_{1}\in R^{64}$$

Where b1 is bias vector and W1 is the weight matrix Also, ReLU (x) is max (0,x) 

#### 5. Dropout

 Dropout is applied to tge 32-dimensional representation: 


(8) $$a_{\mathrm{drop}}=\mathrm{Dropout}(a_{1},p=0.3)$$

#### 6. Output Layer

 The final representation is mapped to a scalar using a sigmoid activation function for binary classification: 


(9) $$y_{\mathrm{pred}}=\sigma (W_{2}a_{\mathrm{drop}}+b_{2}),y_{\mathrm{pred}}\in [0,1]$$

The proposed model begins with an LSTM layer with 64 nodes (represented by Equations 4 and 5), which processes sequences of input data, represented by Equation 3, and return the hidden states. LSTM networks can learn long-term dependencies from sequential input data. Next, a dropout layer, represented by Equation 6, randomly deactivates a defined percentage of neurons during the learning stage. The use of dropout helps minimize overfitting by forcing the model to learn features in a more robust and generalized manner. The output from the LSTM layer is then passed to a fully connected neural layer (Equation 7) with an ReLU activation function. This introduces nonlinearity into the model, enabling it to learn complex relationships by outputting either zero or the input value, whichever is greater. After the dense layer, another dropout layer is applied (as shown in Equation 8) to minimized potential overfitting. Finally, the model concludes with an output layer (Equation 9) consisting of a single neuron with a sigmoid activation function. 

### Performance parameters

 The proposed LSTM-based model was assessed using standard performance metrics. 

 Accuracy: The ratio of accurately classified cases (true positives [TP] and true negatives [TN]) to all evaluated cases, as expressed in Equation 10. 


(10) $$\mathrm{Accuracy}=(\frac{\mathrm{TP}+\mathrm{TN}}{\mathrm{TP}+\mathrm{TN}+\mathrm{FP}+\mathrm{FN}})$$

Precision: The proportion of TP relative to the total predicted positives. It is calculated using Equation 11. 


(11) $$\mathrm{Precision}=(\frac{\mathrm{TP}}{\mathrm{TP}+\mathrm{FP}})$$

Recall: The proportion of TP relative to all actual positives, calculated using Equation 12. Recall is also referred to as sensitivity: 


(12) $$\mathrm{Sensitivity/Recall}=(\frac{\mathrm{TP}}{\mathrm{TP}+\mathrm{FP}})$$

F1 Score: Computed as the harmonic mean of precision and recall, combining both into a single balanced metric. It is calculated as shown in Equation 13. 


(13) $$\mathrm{F1}\mathrm{score}=2\times (\frac{\mathrm{Precision}\times \mathrm{Recall}}{\mathrm{Precision}+\mathrm{Recall}})$$

## RESULTS

 EEG recordings from 27 subjects followed the international 10-20 system and were stored in EDF^17^. The preprocessed EEG signals were subsequently segmented and then fed into the model for MCI *vs.* normal classification. 

 The dataset was relatively small, which could lead to overfitting; therefore, segmentation was applied to increase the number of training samples and improve model robustness. Additionally, cross-validation was employed to ensure more reliable performance estimates. 

 A key concern with small datasets is the risk of overfitting, in which the model may learn patterns specific to the training data rather than generalizable features of MCI. Although segmentation and cross-validation were applied to reduce this risk, it cannot be fully eliminated given the limited cohort size. 

**Figure 5:**
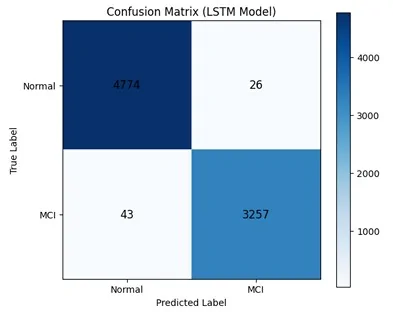
Confusion matrix of the model showing true values *versus* predicted values. Source: Created by the authors.

 The confusion matrix in Figure 5 shows that the proposed LSTM model performs well in distinguishing between normal and MCI EEG segments. The model correctly classified 4,774 normal samples, with only 26 normal samples misclassified as MCI, indicating a very low false-positive rate for MCI. Similarly, 3,257 MCI samples were correctly identified, while 43 MCI samples were incorrectly labeled as normal, reflecting a relatively low false-negative rate. Overall, the high number of correctly classified samples in both classes suggests that the model achieves strong discriminative performance for the normal and MCI categories in this dataset. 

**Figure 6:**
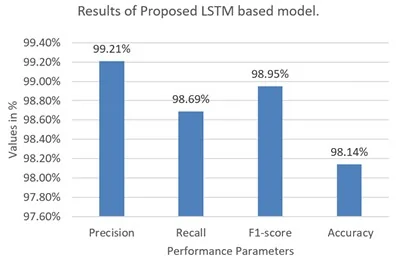
Performance metrics of the proposed long short-term memory-based model, presenting precision, recall, F1-score, and accuracy. Source: Created by the authors.

 The model achieved an accuracy of 98.14% for the binary classification task. It demonstrated a precision of 99.21% and a recall of 98.69%. The model was also evaluated using the F1-score, achieving a value of 98.95%. Figure 6 presents the performance results obtained with the proposed model. 

### Computational environment

 The proposed MCI *vs.* normal binary EEG classification system was implemented and tested on a personal workstation with the following specifications: 12^th^ Gen Intel Core i5-1240P processor @ 1.70 GHz, 16.0 GB RAM (15.7 GB usable), 64-bit Windows 11 Version 23H2 operating system, and 476 GB SSD storage. Training of the computationally intensive DL model was performed using Google Colab with T4 GPU support. All implementations were developed in Python. 

 The 5-fold cross-validation analysis highlights the strong and reliable performance of the proposed framework for distinguishing MCI from normal EEG signals. Table 2 presents the 5-fold cross-validation results for the proposed LSTM-based model. The model achieved an accuracy of 98.01% in Fold 1, whereas a slight decline in performance was observed in Fold 2. Across all folds, the model consistently achieved high values for accuracy, precision, recall, and F1-score, with average values of 97.82, 97.89, 97.80, and 97.84%, respectively. 

**Table 2:** Model evaluation using 5-fold cross-validation

Fold	Accuracy (%)	Precision (%)	Recall (%)	F1-Score (%)
1	98.01	97.67	98.02	97.844687
2	97.82	97.22	98.34	97.7767928
3	97.89	97.92	97.33	97.62410858
4	97.37	98.01	97.74	97.87481379
5	98.02	98.61	97.58	98.09229624
Average	97.822	97.886	97.802	97.84253968

Source: Created by the authors.

## DISCUSSION

 Many researchers have investigated MCI identification using both ML and DL approaches with heterogeneous datasets. The heterogeneity in datasets arises from differences in data acquisition protocols, preprocessing techniques, and the specific cognitive tests used for MCI diagnosis. This diversity makes direct comparisons between studies challenging. However, a general trend indicates that DL approaches, in particular, often outperform conventional ML techniques in terms of performance and generalizability. 

 In this study, each 30-minute EEG recording was divided into shorter 6-second segments, and these segments were then randomly split into training and testing sets, resulting in segment-wise splitting and cross-validation. 

 Based on the 5-fold cross-validation results presented in Table 3 ^14^, the small variation across folds highlights the stability and robustness of the model, suggesting that the learned feature representations are not overly sensitive to data partitioning. The observed difference between the 5-fold cross-validation accuracy (97.82%) and the accuracy obtained from training and testing on the entire dataset (98.14%) is small and expected. These results suggest that the classifier is not only accurate but also balanced in its ability to correctly detect MCI cases while minimizing the misclassification of normal subjects. Importantly, the low variability across folds indicates that the method is stable and not overly dependent on any particular subset of the data. Such robustness is critical in clinical contexts, where consistent performance is required for reliable early detection of MCI. Furthermore, the high recall values demonstrate the model’s effectiveness in reducing false negatives, which is particularly important because failure to identify MCI cases may delay timely intervention. 

**Table 3:** Comparison with existing research works on the same dataset with the long short-term memory approach.

Author	Method	Performance (accuracy%)
Alvi et al.^14^	LSTM (128 nodes)	94.37
Alvi et al.^14^	LSTM (1024 nodes)	96.41
Proposed LSTM based model	LSTM (64 nodes)	98.14

Abbreviations: LSTM, long short-term memory.

Source: Created by the authors.

Table 3 presents studies utilizing the same IUMS dataset of 27 subjects (11 MCI and 16 normal) for model development and that also employed a basic LSTM-based model. A study^14^ implemented LSTM architectures with 128 and 1,024 nodes, achieving accuracies of 94.37 and 96.41%, respectively. The proposed model outperformed these approaches, achieving an accuracy of 98.14%. 

Table 4 ^17-21^ shows the results of studies on MCI detection using the same IUMS dataset but employing different ML-based approaches. 

**Table 4:** Comparison with existing research works on same dataset with different approach.

Author	Method	Performance (accuracy%)
Rabbani et al.^17^	K- Nearest Neighbour classifier (KNN)	88.89
Yin et al.^18^	Support Vector Machine Classifier	96.94
Hadiyoso et al.^19^	K- Nearest Neighbour classifier (KNN)	81.5
Jamaloo et al.^20^	Hidden Markov model	95.9
Ohal et al.^21^	Statistical Features Based SVM model	92.00
Proposed LSTM based model	LSTM (64 nodes)	98.14

Abbreviations: LSTM, long short-term memory; SVM, Support Vector Machine.

Source: Created by the authors.

 There are limited publicly available EEG-based datasets for AD and MCI, and those available are heterogeneous; therefore, fair comparison between different methods remains challenging. 

 In conclusion, this study demonstrated the effective use of DL based on LSTM networks for detecting MCI through EEG signal analysis compared with normal cognitive performance assessment. Early detection of MCI plays a pivotal role in preventing progression toward severe cognitive deterioration associated with AD. 

 By leveraging EEG data, advanced preprocessing techniques, and DL, the proposed framework achieved and accuracy of 98.14%, outperforming previous methodologies. The encouraging results observed suggest that the proposed LSTM framework captures meaningful temporal patterns in EEG signals. This work also compares the results with existing ML-based and DLbased approaches. The findings highlight the potential of LSTM networks for identifying complex temporal behaviors in EEG signals, enabling more reliable and scalable early detection systems. 

 The limitations of this study should be acknowledged. The proposed approach was evaluated using the IUMS EEG dataset consisting of 27 subjects, representing a relatively small cohort. Due to the limited dataset size, the model may be susceptible to overfitting, potentially resulting in optimistic performance estimates. In addition, the evaluation was conducted using a single dataset, which may limit the generalizability of the findings to other populations or EEG datasets collected under different acquisition protocols. Variations in recording conditions, demographic characteristics, and larger sample sizes may influence model performance. Furthermore, segment-wise data splitting with cross-validation was adopted in this study, which may introduce the possibility of data leakage between training and testing sets. 

 Future work should consider subject-wise data splitting and validation using larger and more diverse datasets to further assess the robustness and generalizability of the proposed model. Challenges remain regarding the generalizability of models across multiple populations and the integration of AI-based diagnostic models into real clinical workflows. Addressing these challenges will be necessary to bridge the gap between technological advancement and meaningful clinical implementation. To improve generalizability, future work should therefore focus on extending this analysis to larger, multicenter, and more diverse datasets. Additionally, transfer learning techniques may be explored to adapt the model to different datasets. 

## Data Availability

The datasets generated and/or analyzed during the current study are publicly available at https://misp.mui.ac.ir/en/eeg-data-0

## References

[1] Bolla G, Berente DB, Andrássy A, Zsuffa JA, Hidasi Z, Csibri E (2023). Comparison of the diagnostic accuracy of resting-state fMRI driven machine learning algorithms in the detection of mild cognitive impairment. Sci Rep.

[2] Karapapas C, Goumopoulos C (2021). Mild cognitive impairment detection using machine learning models trained on data collected from serious games. Appl Sci.

[3] Seifallahi M, Galvin JE, Ghoraani B (2024). Detection of mild cognitive impairment using various types of gait tests and machine learning. Front Neurol.

[4] Akl A, Taati B, Mihailidis A (2015). Autonomous unobtrusive detection of mild cognitive impairment in older adults. IEEE Trans Biomed Eng.

[5] Jitsuishi T, Yamaguchi A (2022). Searching for optimal machine learning model to classify mild cognitive impairment (MCI) subtypes using multimodal MRI data. Sci Rep.

[6] Yang D, Hong KS (2021). Quantitative assessment of resting-state for mild cognitive impairment detection: a functional near-infrared spectroscopy and deep learning approach. J Alzheimers Dis.

[7] Veneziani I, Marra A, Formica C, Grimaldi A, Marino S, Quartarone A (2024). Applications of artificial intelligence in the neuropsychological assessment of dementia: a systematic review. J Pers Med.

[8] Hochreiter S, Schmidhuber J (1997). Long short-term memory. Neural Computation.

[9] Sak H, Senior A, Beaufays F (2014). Long short-term memory based recurrent neural network architectures for large vocabulary speech recognition. arXiv.

[10] Malashin I, Tynchenko V, Gantimurov A, Nelyub V, Borodulin A (2024). Applications of Long Short-Term Memory (LSTM) networks in polymeric sciences: a review. Polymers (Basel).

[11] Kashefpoor M, Rabbani H, Barekatain M (2016). Automatic diagnosis of mild cognitive impairment using electroencephalogram spectral features. J Med Signals Sens.

[12] Kim CS, Sun J, Liu D, Wang Q, Paek SG (2024). Notice of removal: removal of ocular artifacts using ICA and adaptive filter for motor imagery-based BCI. IEEE/CAA Journal of Automatica Sinica.

[13] Kim M jae, Youn YC, Paik J (2023). Deep learning-based EEG analysis to classify normal, mild cognitive impairment, and dementia: algorithms and dataset. Neuroimage.

[14] Alvi AM, Siuly S, Wang H (2023). A long short-term memory based framework for early detection of mild cognitive impairment from EEG signals. IEEE Trans Emerg Top Comput Intell.

[15] Faraz M, Khaloozadeh H, Abbasi M (2020). Stock market prediction-by-prediction based on autoencoder long short-term memory networks. 28th Iranian Conference on Electrical Engineering.

[16] Smagulova K, James AP (2020). Overview of long short-term memory neural networks. Modeling and Optimization in Science and Technologies.

[17] Kashefpoor M, Rabbani H, Barekatain M (2019). Supervised dictionary learning of EEG signals for mild cognitive impairment diagnosis. Biomed Signal Process Control.

[18] Yin J, Cao J, Siuly S, Wang H (2019). An integrated MCI detection framework based on spectral-temporal analysis. Int J Autom Comput.

[19] Hadiyoso S, Cynthia CLFAR, Mengko TLER, Zakaria H (2019). 2nd International Conference on Bioinformatics, Biotechnology and Biomedical Engineering (BioMIC)-Bioinformatics and Biomedical Engineering.

[20] Jamaloo F, Mikaeili M, Noroozian M (2020). Multi metric functional connectivity analysis based on continuous hidden Markov model with application in early diagnosis of Alzheimer’s disease. Biomed Signal Process Control.

[21] Ohal HS, Mantri S (2024). Exploring EEG-Based biomarkers for improved early alzheimer’s disease detection: a feature-based approach utilizing machine learning. Measur Sens.

